# Supplementary material for: Genetic Variants of HNF4A, WFS1, DUSP9, FTO, and ZFAND6 Genes Are Associated with Prediabetes Susceptibility and Inflammatory Markers in the Saudi Arabian Population
Source: Genes (Basel). 2023 Feb 21;14(3):536. doi: 10.3390/genes14030536 (PMC10048403; doi:10.3390/genes14030536)
Supplement: Supplementary file 1 [file genes-14-00536-s001.zip › genes-2194425-supplementary.pdf]

Table S1. Association between SNPs and Pre-diabetes status 1

SNPs		Control	Prediabetes	Unadjusted		Adjusted	
				OR (95%CI)	*p*-value	OR (95%CI)	*p*-value
rs7903146	CC	369 (46.8)	153 (46.5)	Reference		Reference	
	TC	324 (41.1)	143 (43.5)	1.06 (0.81 - 1.40)	0.65	1.02 (0.77 - 1.34)	0.90
	TT	96 (12.2)	33 (10.0)	0.83 (0.53 - 1.28)	0.40	0.89 (0.57 - 1.39)	0.60
rs5015480	CC	403 (51.0)	170 (51.4)	Reference		Reference	
	TC	318 (40.3)	122 (36.9)	0.91 (0.69 - 1.20)	0.50	0.88 (0.66 - 1.16)	0.36
	TT	69 (8.7)	39 (11.8)	1.34 (0.87 - 2.06)	0.18	1.32 (0.85 - 2.06)	0.22
rs12779790	AA	559 (70.8)	219 (67.0)	Reference		Reference	
	GA	202 (25.6)	95 (29.1)	1.20 (0.90 - 1.60)	0.22	1.23 (0.91 - 1.65)	0.18
	GG	29 (3.7)	13 (4.0)	1.14 (0.58 - 2.24)	0.69	1.15 (0.58 - 2.27)	0.70
rs10923931	GG	625 (79.2)	258 (78.4)	Reference		Reference	
	TG	153 (19.4)	65 (19.8)	1.03 (0.74 - 1.42)	0.86	0.98 (0.70 - 1.36)	0.90
	TT	11 (1.4)	6 (1.8)	1.32 (0.48 - 3.61)	0.59	1.18 (0.42 - 3.30)	0.75
rs10440833	TT	485 (61.7)	200 (60.4)	Reference		Reference	
	TA	255 (32.4)	107 (32.3)	1.02 (0.77 - 1.35)	0.90	1.02 (0.77 - 1.36)	0.88
	AA	46 (5.9)	24 (7.3)	1.27 (0.75 - 2.13)	0.38	1.31 (0.76 - 2.23)	0.33
rs11899863	GG	720 (90.9)	309 (93.1)	Reference		Reference	
	GA	71 (9.0)	21 (6.3)	0.69 (0.42 - 1.14)	0.15	0.69 (0.41 - 1.16)	0.16
	AA	1 (0.1)	2 (0.6)	4.66 (0.42 - 51.58)	0.21	4.68 (0.41 - 54.00)	0.22
rs13081389	AA	716 (90.7)	306 (93.3)	Reference		Reference	
	GA	68 (8.6)	21 (6.4)	0.72 (0.44 - 1.20)	0.21	0.76 (0.45 - 1.27)	0.29
	GG	5 (0.6)	1 (0.3)	0.47 (0.05 - 4.02)	0.49	0.51 (0.06 - 4.48)	0.54
rs3802177	GG	580 (73.4)	248 (75.2)	Reference		Reference	
	GA	187 (23.7)	77 (23.3)	0.96 (0.71 - 1.31)	0.81	0.91 (0.67 - 1.25)	0.56
	AA	23 (2.9)	5 (1.5)	0.51 (0.19 - 1.35)	0.18	0.51 (0.19 - 1.36)	0.18
rs849134	TT	297 (37.5)	121 (36.6)	Reference		Reference	
	TC	367 (46.3)	145 (43.8)	0.97 (0.73 - 1.29)	0.83	1.02 (0.76 - 1.36)	0.92
	CC	128 (16.2)	65 (19.6)	1.25 (0.86 - 1.80)	0.24	1.33 (0.91 - 1.93)	0.14
rs5215	TT	560 (70.6)	228 (69.1)	Reference		Reference	
	TC	197 (24.8)	92 (27.9)	1.15 (0.86 - 1.54)	0.36	1.16 (0.86 - 1.57)	0.32
	CC	36 (4.5)	10 (3.0)	0.68 (0.33 - 1.40)	0.30	0.66 (0.32 - 1.37)	0.27
rs1470579	AA	364 (46.0)	146 (44.0)	Reference		Reference	
	CA	296 (37.4)	119 (35.8)	1.00 (0.75 - 1.33)	0.99	1.02 (0.76 - 1.36)	0.92
	CC	131 (16.6)	67 (20.2)	1.28 (0.90 - 1.81)	0.18	1.28 (0.89 - 1.85)	0.18
rs6795735	TT	441 (55.8)	181 (54.8)	Reference		Reference	
	TC	297 (37.6)	119 (36.1)	0.98 (0.74 - 1.28)	0.86	0.95 (0.72 - 1.25)	0.71
	CC	52 (6.6)	30 (9.1)	1.41 (0.87 - 2.28)	0.17	1.44 (0.88 - 2.37)	0.14
rs1387153	CC	433 (54.9)	167 (51.1)	Reference		Reference	
	TC	293 (37.2)	126 (38.5)	1.11 (0.85 - 1.47)	0.44	1.10 (0.83 - 1.45)	0.52
	TT	62 (7.9)	34 (10.4)	1.42 (0.90 - 2.24)	0.13	1.45 (0.91 - 2.31)	0.12
rs243021	TT	323 (40.8)	117 (35.7)	Reference		Reference	

	TC	334 (42.2)	157 (47.9)	1.30 (0.98 - 1.72)	0.07	1.30 (0.97 - 1.74)	0.08
	CC	135 (17.0)	54 (16.5)	1.10 (0.76 - 1.61)	0.61	1.08 (0.73 - 1.59)	0.69
	AA	243 (30.9)	101 (30.8)	Reference		Reference	
rs7578326	GA	377 (47.9)	165 (50.3)	1.05 (0.78 - 1.42)	0.73	1.05 (0.77 - 1.41)	0.77
	GG	167 (21.2)	62 (18.9)	0.89 (0.62 - 1.30)	0.55	0.92 (0.63 - 1.35)	0.67
	AA	350 (44.4)	129 (38.9)	Reference		Reference	
rs4457053	GA	327 (41.5)	161 (48.5)	1.34 (1.01 - 1.76)	0.04	1.31 (0.99 - 1.74)	0.06
	GG	111 (14.1)	42 (12.7)	1.03 (0.68 - 1.54)	0.90	1.04 (0.68 - 1.58)	0.87
	GG	277 (35.2)	118 (35.6)	Reference		Reference	
rs972283	GA	382 (48.6)	149 (45.0)	0.92 (0.69 - 1.22)	0.55	0.90 (0.67 - 1.20)	0.46
	AA	127 (16.2)	64 (19.3)	1.18 (0.82 - 1.71)	0.37	1.18 (0.81 - 1.72)	0.40
	AA	229 (29.0)	92 (27.8)	Reference		Reference	
rs896854	GA	351 (44.5)	165 (49.8)	1.17 (0.86 - 1.59)	0.31	1.23 (0.90 - 1.67)	0.20
	GG	209 (26.5)	74 (22.4)	0.88 (0.62 - 1.26)	0.49	0.93 (0.64 - 1.34)	0.70
	CC	774 (97.9)	321 (97.0)	Reference		Reference	
rs13292136	TC	16 (2.0)	10 (3.0)	1.51 (0.68 - 3.36)	0.32	1.39 (0.62 - 3.12)	0.43
	TT	1 (0.1)	0 (0.0)	0.80 (0.03 - 19.7)	0.89	0.00 (0.00 - 0.00)	0.99
	TT	250 (31.6)	96 (29.1)	Reference		Reference	
rs231362	TC	362 (45.7)	169 (51.2)	1.22 (0.90 - 1.64)	0.20	1.22 (0.90 - 1.66)	0.19
	CC	180 (22.7)	65 (19.7)	0.94 (0.65 - 1.36)	0.74	0.90 (0.62 - 1.31)	0.58
	TT	760 (96.0)	312 (94.5)	Reference		Reference	
rs1552224	TG	30 (3.8)	18 (5.5)	1.46 (0.80 - 2.66)	0.21	1.52 (0.83 - 2.79)	0.18
	GG	2 (0.3)	0 (0.0)	0.48 (0.02 - 10.17)	0.64	0.00 (0.00 - 0.00)	0.99
	TT	572 (72.8)	233 (70.6)	Reference		Reference	
rs7957197	TA	194 (24.7)	83 (25.2)	1.05 (0.78 - 1.42)	0.75	1.02 (0.75 - 1.38)	0.91
	AA	20 (2.5)	14 (4.2)	1.72 (0.85 - 3.46)	0.13	1.82 (0.90 - 3.70)	0.10
	GG	244 (30.8)	114 (34.4)	Reference		Reference	
rs11634397	GA	402 (50.8)	142 (42.9)	0.76 (0.56 - 1.01)	0.06	0.75 (0.56 - 1.01)	0.06
	AA	146 (18.4)	75 (22.7)	1.10 (0.77 - 1.57)	0.60	1.06 (0.74 - 1.53)	0.75
	AA	229 (29.1)	80 (24.6)	Reference		Reference	
rs8042680	CA	347 (44.1)	154 (47.4)	1.27 (0.92 - 1.75)	0.14	1.19 (0.86 - 1.64)	0.30
	CC	211 (26.8)	91 (28.0)	1.23 (0.87 - 1.76)	0.24	1.21 (0.84 - 1.73)	0.31
	AA	628 (79.5)	282 (85.2)	Reference		Reference	
rs5945326	GA	122 (15.4)	30 (9.1)	0.55 (0.36 - 0.84)	0.005	0.60 (0.39 - 0.92)	0.02
	GG	40 (5.1)	19 (5.7)	1.06 (0.60 - 1.86)	0.84	1.03 (0.58 - 1.83)	0.92
	TT	247 (31.5)	97 (29.6)	Reference		Reference	
rs163184	TG	359 (45.7)	161 (49.1)	1.14 (0.85 - 1.54)	0.39	1.18 (0.87 - 1.61)	0.28
	GG	179 (22.8)	70 (21.3)	1.00 (0.69 - 1.43)	0.98	1.04 (0.72 - 1.51)	0.82
	GG	259 (32.9)	120 (36.5)	Reference		Reference	
rs4430796	GA	366 (46.4)	146 (44.4)	0.86 (0.64 - 1.15)	0.31	0.85 (0.63 - 1.14)	0.28
	AA	163 (20.7)	63 (19.1)	0.83 (0.58 - 1.20)	0.33	0.84 (0.58 - 1.21)	0.34
	GG	485 (61.6)	231 (70.0)	Reference		Reference	
rs4812829	GA	273 (34.7)	83 (25.2)	0.64 (0.48 - 0.85)	0.003	0.64 (0.47 - 0.86)	0.003
	AA	29 (3.7)	16 (4.8)	1.16 (0.62 - 2.18)	0.65	1.17 (0.62 - 2.22)	0.63
rs1802295	CC	369 (47.5)	162 (49.4)	Reference		Reference	

	TC	325 (41.8)	137 (41.8)	0.96 (0.73 - 1.26)	0.77	0.95 (0.72 - 1.26)	0.73
	TT	83 (10.7)	29 (8.8)	0.80 (0.50 - 1.26)	0.33	0.85 (0.53 - 1.35)	0.48
	GG	340 (43.1)	134 (40.7)	Reference		Reference	
rs7178572	GA	336 (42.6)	157 (47.7)	1.19 (0.90 - 1.56)	0.23	1.21 (0.91 - 1.60)	0.19
	AA	112 (14.2)	38 (11.6)	0.86 (0.57 - 1.31)	0.48	0.85 (0.56 - 1.31)	0.47
	AA	493 (62.5)	202 (61.6)	Reference		Reference	
rs2028299	CA	255 (32.3)	103 (31.4)	0.99 (0.74 - 1.31)	0.92	0.96 (0.72 - 1.28)	0.80
	CC	41 (5.2)	23 (7.0)	1.37 (0.80 - 2.34)	0.25	1.42 (0.83 - 2.45)	0.20
	AA	335 (42.7)	155 (47.0)	Reference		Reference	
rs3923113	CA	346 (44.1)	139 (42.1)	0.87 (0.66 - 1.14)	0.31	0.85 (0.65 - 1.13)	0.27
	CC	103 (13.1)	36 (10.9)	0.76 (0.49 - 1.15)	0.20	0.75 (0.49 - 1.16)	0.20
	CC	573 (72.9)	242 (73.3)	Reference		Reference	
rs16861329	TC	192 (24.4)	80 (24.2)	0.99 (0.73 - 1.33)	0.93	0.98 (0.72 - 1.33)	0.90
	TT	21 (2.7)	8 (2.4)	0.90 (0.39 - 2.06)	0.81	0.89 (0.38 - 2.07)	0.79
	GG	424 (53.9)	172 (52.6)	Reference		Reference	
	GC	284 (36.1)	129 (39.4)	1.12 (0.85 - 1.47)	0.42	1.08 (0.82 - 1.43)	0.59
rs1531343	CC	79 (10.0)	26 (8.0)	0.81 (0.50 - 1.31)	0.39	0.74 (0.45 - 1.22)	0.24
	TT	222 (28.4)	129 (39.0)	Reference		Reference	
rs1801214	TC	405 (51.7)	142 (42.9)	0.60 (0.45 - 0.81)	0.001	0.60 (0.44 - 0.80)	0.001
	CC	156 (19.9)	60 (18.1)	0.66 (0.46 - 0.96)	0.03	0.67 (0.46 - 0.98)	0.04
	GG	446 (56.8)	188 (57.5)	Reference		Reference	
rs10965250	GA	291 (37.1)	118 (36.1)	0.96 (0.73 - 1.26)	0.78	0.97 (0.73 - 1.28)	0.82
	AA	48 (6.1)	21 (6.4)	1.04 (0.60 - 1.78)	0.89	1.09 (0.63 - 1.89)	0.75
	CC	332 (42.2)	124 (37.6)	Reference		Reference	
rs11642841	CA	349 (44.3)	144 (43.6)	1.10 (0.83 - 1.47)	0.49	1.08 (0.81 - 1.45)	0.58
	AA	106 (13.5)	62 (18.8)	1.57 (1.08 - 2.28)	0.02	1.50 (1.02 - 2.21)	0.04

Note: Data presented as N (%) and Odds ratio (95% CI) obtained from logistic regression; Adjusted indicates results adjusted for covariates i.e. age, gender and BMI; *p*-value < 0.05 was considered significant.

Table S2: Hardy-Weinberg Equilibrium

SNPs		Control	Prediabetes	HWE Test *p*-value	Unadjusted		Adjusted	
					OR (95%CI)	*p*-value	OR (95%CI)	*p*-value
rs11634397	GG	244 (30.8)	114 (34.4)		Reference		Reference	
	GA	402 (50.8)	142 (42.9)	0.41	0.76 (0.56 - 1.01)	0.06	0.75 (0.56 - 1.01)	0.05
	AA	146 (18.4)	75 (22.7)		1.10 (0.77 - 1.57)	0.60	1.06 (0.74 - 1.53)	0.75
rs5945326	AA	628 (79.5)	282 (85.2)		Reference		Reference	
	GA	122 (15.4)	30 (9.1)	<0.001	0.55 (0.36 - 0.84)	0.005	0.60 (0.39 - 0.92)	0.01
	GG	40 (5.1)	19 (5.7)		1.06 (0.60 - 1.86)	0.84	1.03 (0.58 - 1.83)	0.92
rs4812829	GG	485 (61.6)	231 (70.0)		Reference		Reference	
	GA	273 (34.7)	83 (25.2)	0.22	0.64 (0.48 - 0.85)	0.003	0.64 (0.47 - 0.86)	<0.01
	AA	29 (3.7)	16 (4.8)		1.16 (0.62 - 2.18)	0.65	1.17 (0.62 - 2.22)	0.63
rs1801214	TT	222 (28.4)	129 (39.0)		Reference		Reference	
	TC	405 (51.7)	142 (42.9)	0.24	0.60 (0.45 - 0.81)	0.001	0.60 (0.44 - 0.80)	<0.01
	CC	156 (19.9)	60 (18.1)		0.66 (0.46 - 0.96)	0.03	0.67 (0.46 - 0.98)	0.04
rs11642841	CC	332 (42.2)	124 (37.6)		Reference		Reference	
	CA	349 (44.3)	144 (43.6)	0.33	1.10 (0.83 - 1.47)	0.49	1.08 (0.81 - 1.45)	0.58
	AA	106 (13.5)	62 (18.8)		1.57 (1.08 - 2.28)	0.02	1.50 (1.02 - 2.21)	0.03

Note: Data presented as N (%) and Odds ratio (95% CI) obtained from logistic regression; Adjusted indicates results adjusted for covariates i.e. age, gender and BMI; *p*-value < 0.05 was considered significant.
